# *IDH* Mutations in Glioma: Double-Edged Sword in Clinical Applications?

**DOI:** 10.3390/biomedicines9070799

**Published:** 2021-07-10

**Authors:** Alisan Kayabolen, Ebru Yilmaz, Tugba Bagci-Onder

**Affiliations:** 1 Brain Cancer Research and Therapy Lab, Koç University School of Medicine, 34450 Istanbul, Turkey; akayabolen@ku.edu.tr (A.K.); eyilmaz20@ku.edu.tr (E.Y.); 2 Koç University Research Center for Translational Medicine (KUTTAM), 34450 Istanbul, Turkey

**Keywords:** isocitrate dehydrogenase (IDH), mutations, glioma, glioblastoma, therapeutics, clinical trials

## Abstract

Discovery of point mutations in the genes encoding isocitrate dehydrogenases (IDH) in gliomas about a decade ago has challenged our view of the role of metabolism in tumor progression and provided a new stratification strategy for malignant gliomas. IDH enzymes catalyze the conversion of isocitrate to alpha-ketoglutarate (α-KG), an intermediate in the citric acid cycle. Specific mutations in the genes encoding IDHs cause neomorphic enzymatic activity that produces D-2-hydroxyglutarate (2-HG) and result in the inhibition of α-KG-dependent enzymes such as histone and DNA demethylases. Thus, chromatin structure and gene expression profiles in *IDH*-mutant gliomas appear to be different from those in *IDH*-wildtype gliomas. *IDH* mutations are highly common in lower grade gliomas (LGG) and secondary glioblastomas, and they are among the earliest genetic events driving tumorigenesis. Therefore, inhibition of mutant IDH enzymes in LGGs is widely accepted as an attractive therapeutic strategy. On the other hand, the metabolic consequences derived from *IDH* mutations lead to selective vulnerabilities within tumor cells, making them more sensitive to several therapeutic interventions. Therefore, instead of shutting down mutant IDH enzymes, exploiting the selective vulnerabilities caused by them might be another attractive and promising strategy. Here, we review therapeutic options and summarize current preclinical and clinical studies on *IDH*-mutant gliomas.

## 1. Introduction

**Figure 1:**
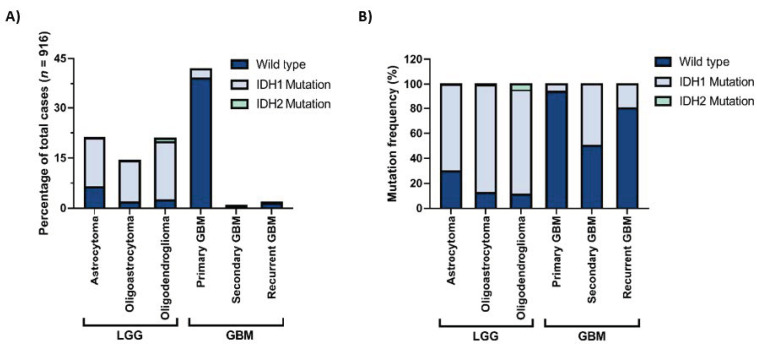
*IDH1* and *IDH2* mutation frequencies in gliomas with different histological grades. (**A**) Percentages of *IDH1* and *IDH2* mutations in different histological grades, all glioma cases. (**B**) Distribution of *IDH1* and *IDH2* mutations in lower-grade gliomas (LGG) and glioblastomas (GBM). The figure is generated with data obtained from the GlioVis portal [3].

Gliomas are the most common central nervous system (CNS) tumors in adults [1]. They can be classified based on the cellular origin of the tumor into astrocytomas, oligodendrogliomas, or ependymomas or on the aggressiveness of the tumor into grade I–IV gliomas. These classifications are determined by histopathological analysis which may sometimes be highly difficult and subjective. In 2016, the World Health Organization (WHO) published a report offering a classification of CNS tumors based on both histological and molecular features [2]. In this report, one of the most important diagnostic markers of diffuse glioma is the status of the IDH mutation. Accordingly, both oligodendrogliomas and astrocytomas, either lower-grade (grade II or III) or glioblastomas (grade IV), are separated into *IDH*-wildtype and *IDH*-mutant. While the most malignant subtype of gliomas, glioblastoma (GBM), can appear de novo and be called primary GBM, it may also result from progression from lower-grade astrocytomas and be called secondary GBM. Indeed, lower-grade gliomas (LGG) and primary GBMs make up most of the glioma cases (Figure 1A). Among these, *IDH* mutations are markedly observed in LGGs and secondary GBMs (Figure 1B) and are among the early genetic events in tumor progression.

Isocitrate dehydrogenases (IDH) are enzymes that convert isocitrate to alpha-ketoglutarate (α-KG, 2-oxoglutarate, 2-OG). In humans, *IDH1*, *IDH2,* and *IDH3* genes express three isoforms of the IDH enzyme, which all have significant functions in metabolic reactions. IDH1 is found in the cytoplasm and peroxisome, while IDH2 and IDH3 are in the mitochondrial matrix. Although IDH1 and IDH2 have different locations, they are both isoenzymes and catalyze the conversion of isocitrate to 2-OG while using nicotinamide adenine dinucleotide phosphate (NADP^+^) as a cofactor to produce NADPH as a byproduct [4]. The cytosolic NADPH is an important antioxidant and has major roles in lipid metabolism [5]. On the other hand, 2-OG is used as a cofactor by 2-OG-dependent dioxygenases. The best-known 2-OG-dependent dioxygenases in the nucleus are Jumonji-C (JmjC) domain-containing enzymes, which are histone lysine demethylases (KDMs), and ten–eleven translocation (TET) enzymes, which are DNA demethylases. On the other hand, prolyl hydroxylase domain (PHD)-containing enzymes are examples of cytosolic dioxygenases, which have a broad range of metabolic functions [6]. IDH3 similarly catalyzes the formation of 2-OG from isocitrate, yet by utilizing NAD^+^ as its cofactor [7], and produces NADH used for adenosine triphosphate (ATP) generation in the electron transport chain. There are also other important differences between IDH isoforms. IDH1 and IDH2 function as homodimers and catalyze reversible reactions [8], while IDH3 functions as a heterotetramer with its different subunits and catalyzes an irreversible reaction [9].

The mutations observed in IDH enzymes in gliomas are mainly in cytosolic IDH1 and mitochondrial IDH2, most frequently at codons R132 and R172, respectively [10]. These mutations cause IDH1 and IDH2 to gain a neomorphic enzymatic function, by which they convert 2-OG produced by the wildtype IDH enzyme into D-2-hydroxyglutarate (2-HG) that is thought to be an oncometabolite [11]; it acts as an antagonist of 2-OG and therefore inhibits the enzymatic activities of 2-OG dependent enzymes, KDMs, or TET family enzymes [12,13]. Therefore, *IDH* mutations result in hypermethylated DNA and histone profile, which is considered to be among the major mechanisms behind tumorigenesis [14,15].

In vivo glioma models, which are extremely useful tools to study tumorigenesis [16], indicated that *IDH* mutations by themselves, despite being early events, are not sufficient to drive tumorigenesis, making the involvement of other molecular players necessary [17]. For example, telomerase reverse transcriptase (*TERT*) promoter, *Drosophila* homolog of capicua (*CIC*) gene, far upstream element-binding protein 1 gene (*FUBP1*) mutations associated with chromosome 1p/19q codeletion are diagnostic markers of oligodendrogliomas [18,19]. Mutations in the tumor protein 53 (*TP53*) gene and alterations in the *ATRX* gene along with chromosome 1p/19q non-codeletion are characteristic determinants of astrocytic lineage gliomas [20,21]. These additional mutations may either be early events or acquired during tumor progression. Some gliomas have been named “triple-negative,” referring to the ones that have an *IDH*-wildtype profile without the *p53* mutation or chromosome 1p/19q codeletion [22]. These gliomas may have *EGFR* alterations, which are also considered independent drivers of gliomagenesis [23], *TERT* promoter mutations, chromosome 7 gain, and chromosome 10 loss [20]. Secondary GBMs with *IDH1* mutations are mostly developed from grade II gliomas, whereas secondary GBMs lacking *IDH1* mutations are progressed from grade III gliomas via additional genetic, epigenetic, or chromosomal alterations making them more aggressive [24,25]. Furthermore, grade IV gliomas located along the midline may harbor the *H3 K27M* mutation that causes a global decrease in H3K27 trimethylation [26].

**Figure 2:**
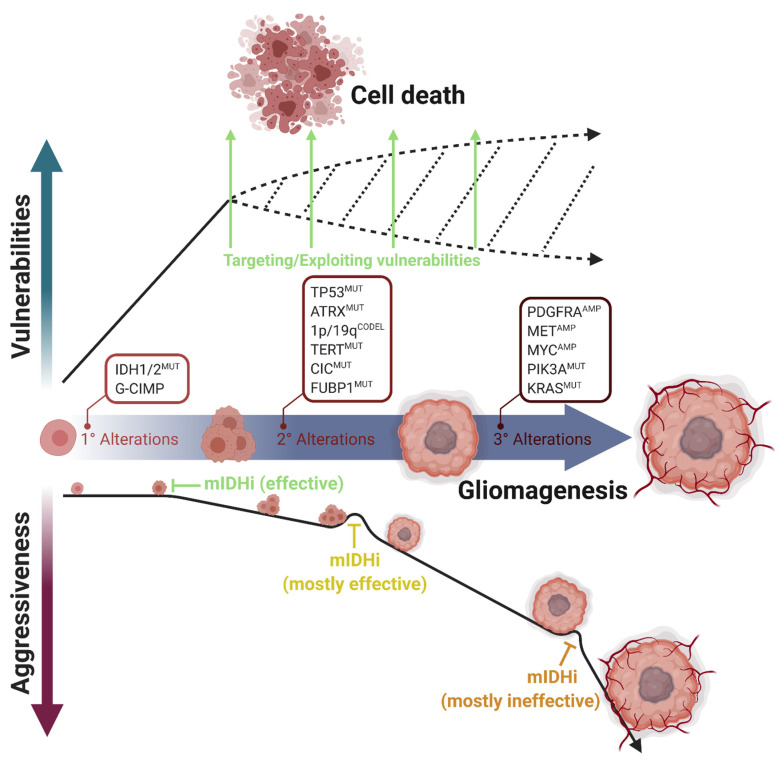
Multistep progression of *IDH*-mutant gliomas and acquisition of selective vulnerabilities. *IDH1/2* mutations are the primary alterations during gliomagenesis. Based on the cell of origin, secondary alterations promote this process differently. *TP53* and *ATRX* mutations are the marker alterations of astrocytomas; *CIC*, *FUBP1*, and *TERT* mutations and 1p/19q codeletions are the marker alterations of oligodendrogliomas. However, tertiary alterations such as *PDGFRA*, *MET*, or *MYC* amplifications or *PIK3A* and *KRAS* mutations increase tumor aggressiveness and mostly render tumor progression independent of *IDH* mutations. Mutant IDH inhibitors (mIDHi) are mostly effective in LGGs, in which only the primary and secondary genetic alterations occur. However, mIDHi are mostly ineffective in secondary glioblastomas, in which tertiary alterations occurred. In parallel, most vulnerabilities appear directly upon *IDH* mutations. Therefore, therapeutic approaches targeting cellular vulnerabilities are promising for *IDH*-mutant gliomas. Figure created with BioRender.com.

Several studies focusing on clonal evolution in tumor samples reveal that *IDH* mutations appear early in oncogenesis and that they are accepted as driver mutations in LGGs [27,28,29]. Therefore, *IDH* mutations provide attractive intervention points and inhibitors of mutant IDH enzymes are considered prime therapeutic candidates [30,31,32]. While this approach is very promising, it imposes several challenges due to the unconventional phenotypes of *IDH*-mutant tumor cells. For example, it is known that *IDH*-mutant cells grow slower than the wildtype ones possibly because they have an altered metabolic profile due to the impaired TCA cycle [33], inhibition of mTOR and the ATP synthase [34], and changes in the expression of the LDHA enzyme [35]. There is an accumulating number of studies examining such specific vulnerabilities of *IDH*-mutant gliomas, including ours [36]. Therefore, exploiting the weaknesses of *IDH*-mutant gliomas and targeting them based on these deficiencies might be an equally, if not more, promising therapeutic strategy (Figure 2). Indeed, the survival benefit received from some of the existing mutant IDH inhibitors was minimal [37,38] in mouse glioma models. The results gained with one of the mutant IDH inhibitors, AGI-5198, were contradictory. While it led to the regression of growth of an *IDH1^R132H^* glioma in one report [30], AGI-5198 did not affect tumor growth despite complete elimination of 2-HG levels [39]. Additionally, these inhibitors may interfere with the efficacy of conventional chemotherapy and radiotherapy [40,41]. Therefore, given the “double-edged sword” nature of *IDH*-mutant gliomas, more systematic research efforts are required to determine the best targeted therapeutic strategy and uncover novel targets for these tumors. In this review, we present an overview of the state-of-the-art of the therapeutic approaches applied in *IDH*-mutant gliomas and provide a summary of both preclinical studies and clinical trials. 

## 2. Potential Therapeutic Approaches 

**Figure 3:**
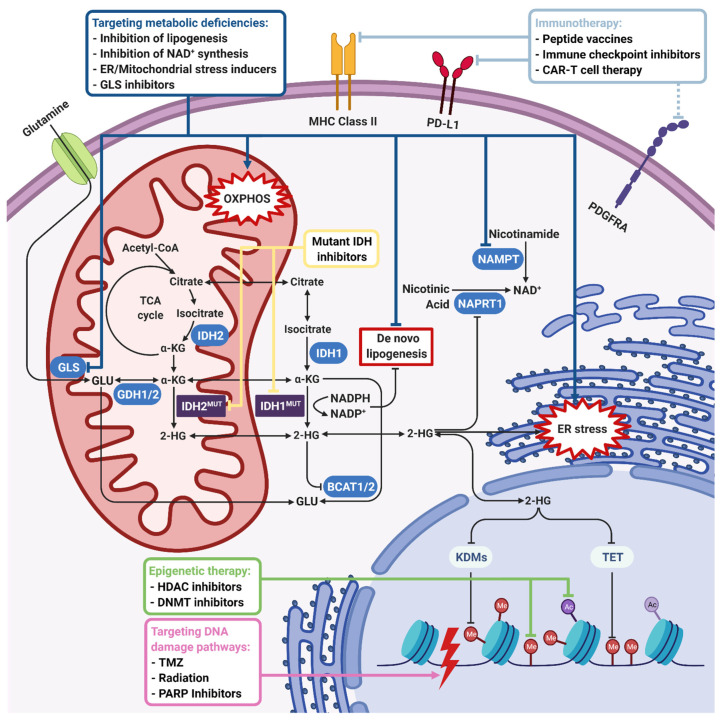
Therapeutic approaches for *IDH*-mutant gliomas. IDH1/2 mutations induce 2-HG accumulation and NADPH depletion, which lead to global metabolic and epigenetic changes in tumor cells. The first-line therapeutic strategy is using mutant *IDH1/2* inhibitors to reverse an *IDH* mutation-induced phenotype. On the other hand, 2-HG is shown to inhibit many 2-OG-dependent dioxygenases such as KDMs, TETs, BCATs, COX, and C-4PH enzymes. NADPH depletion inhibits de novo lipogenesis and causes oxidative stress. Inhibition of KDMs and TETs leads to histone and DNA hypermethylation, which makes targeting epigenetic enzymes a therapeutic option. Upon inhibition of BCATs, cells become dependent on GLS for glutamate production, which is highly important for tumor cell growth. Therefore, GLS inhibition is another therapeutic option. Inhibition of COX, which has an important role in electron transport chain and induction of oxidative stress, renders *IDH*-mutant cells sensitive to mitochondrial stress inducers. On the other hand, 2-HG-induced hypermethylation phenotype results in the repression of the *NAPRT1* gene, which is responsible for NAD+ biosynthesis. Therefore, inhibition of NAMPT responsible for the salvage NAD+ synthesis pathway is another therapeutic option. Depletion of the NAD+ level, 2-HG-dependent inhibition of KDM4A/B, and hypermethylation of the *MGMT* promoter also inhibit DNA damage response, rendering *IDH*-mutant cells sensitive to DNA damage inducers like TMZ, irradiation, or PARP inhibitors. Finally, as the specific IDH1^R132H^ mutation creates an immunogenic epitope, immunotherapeutic approaches including peptide vaccines, immune checkpoint inhibitors and CAR-T cell therapy emerge as other therapeutic strategies. Dark blue, green, purple, and light blue boxes and lines indicate therapeutic approaches targeting metabolic, epigenetic, DNA damage, and immunogenic pathways, respectively. Yellow arrows indicate mutant IDH inhibition directly. Black lines with an arrowhead indicate activated cellular pathways and blunt-end arrows indicate inhibition. 2-HG, 2-hydroxyglutarate; BCAT, branched-chain amino acid (BCAA) aminotransferase; C-4PH, collagen prolyl-4-hydroxylase; COX, cytochrome C oxidase; GLS, glutaminase; KDM, lysine demethylase; MGMT, O^6^-methylguanine DNA methyltransferase; NAD, nicotinamide adenine dinucleotide; NADPH, nicotinamide adenine dinucleotide phosphate (reduced); NAMPT, nicotinamide phosphoribosyltransferase; NAPRT1, nicotinate phosphoribosyltransferase; TET, ten–eleven translocation enzymes; TMZ, temozolomide. Figure created with BioRender.com.

There are several therapeutic approaches either utilizing mutant IDH inhibitors or targeting the specific vulnerabilities of *IDH*-mutant gliomas as explained below (Figure 3). Indeed, there are accompanying clinical trials that either use mutant IDH inhibitors alone, target different pathways that are altered in *IDH*-mutant gliomas, such as DNA damage pathways or epigenetic pathways, or test the effects of combinatorial approaches in *IDH*-mutant tumors.

### 2.1. Mutant IDH Inhibitors

*IDH* mutation is accepted as one of the earliest events in tumorigenesis in gliomas, acute myeloid leukemia (AML) and some other cancer types. Therefore, it is generally the only mutation found homogenously in tumors and preserved in the recurrences as well [27,28,29]. Based on this, mutant IDH enzymes are considered perfect candidates to target leading to the development of mutant IDH-specific inhibitors [30,31,32,38].

In 2013, AGI-5198, the first published inhibitor of mutant IDH1 enzyme, and AGI-6780, the first published inhibitor of the mutant IDH2 enzyme, were shown to be effective against glioma and leukemia cells in vitro [30,31] Inhibition of 2-HG accumulation by these molecules induced differentiation of tumor cells and slowed down the in vivo tumor growth. Many other specific inhibitors targeting mutant IDH1 or IDH2, or pan-inhibitors were developed afterwards [32,38].

Some of these inhibitors were also shown to be effective in clinical trials, and AG-221 (enasidenib) in 2017 and AG-120 (ivosidenib) in 2018 were approved by the FDA for treatment of relapsed or refractory AML with *IDH2* or *IDH1* mutations, respectively [42,43]. Both, together with some new inhibitors, are also included in ongoing clinical trials of gliomas. Moreover, using a noninvasive 3D MRS imaging technique, it was shown that one of these inhibitors, IDH305, reduced 2-HG levels by 70% in a phase I clinical trial [44]. Controversially, some later studies indicated that mutant IDH inhibitors were efficient in preventing 2-HG accumulation, but they failed to reverse global DNA or the histone hypermethylation phenotype and had no significant effect on the growth of glioma cells [45,46,47]. It was even reported that long-term treatment with mutant IDH inhibitors accelerated cell growth and shortened the in vivo survival [45]. Moreover, some studies suggested that mutant IDH inhibitors may interfere with therapeutic approaches targeting mutant IDH-caused vulnerabilities [36]. Therefore, even though mutant IDH enzymes create favorable conditions for tumorigenesis and selection of aggressive mutations, their inhibition may not always be effective in a tumor that has completed its stages of development.

These conflicting results might be related with the role of IDH mutations in tumor growth. Mutations in *IDH1* or *IDH2* are known to be drivers of tumorigenesis in both gliomas and AML [27,28,29]. However, as more aggressive mutations are acquired in later stages of glioma, the *IDH1* mutation has been shown to be converted to a passenger mutation which renders glioma cells to proliferate in a mutant IDH-independent manner [48]. On the other hand, the *IDH2* mutation is mostly required for leukemia cell proliferation and tumor growth [49]. This difference may explain the better effects of mutant IDH inhibitors in AML compared to gliomas. Considering that, it may be hypothesized that mutant IDH inhibitors might be more effective in *IDH*-mutant glioma patients who do not have aggressive tertiary mutations (Figure 2). Clinical trials regarding this difference would give valuable information on this hypothesis.

Still, for both *IDH*-mutant gliomas and other tumor types, there are ongoing clinical trials with mutant IDH inhibitors applied individually **(**Table 1**)** or in combination with other treatment options (Table 2).

### 2.2. Targeting Metabolic Deficiencies

Tumor cells need a high amount of energy and biosynthetic precursors to mediate continuous cell division. For this reason, significant changes are observed in the metabolic activities of tumors. Otto Warburg was one of the first scientists, who observed these metabolic changes in tumor cells. He found that tumor cells mainly use glycolysis to produce energy instead of oxidative phosphorylation even in the presence of oxygen and produce lactic acid by fermentation [55]. On the other hand, cellular metabolism consists of many complex biochemical reactions that are in a well-adjusted balance. Disruption of this balance mostly leads to metabolic dependencies, which can be targeted to induce synthetic lethality [56].

IDH enzymes have many critical roles in cellular metabolism. They catalyze the reaction of isocitrate to the α-KG (2-OG) conversion, which is one of the rate-limiting steps in the TCA cycle, also important for the biosynthesis of many precursors. Therefore, besides driving tumorigenesis, mutations in the *IDH1* or *IDH2* genes lead to the disruption of various metabolic pathways, which generates vulnerabilities to specific treatments. Targeting these vulnerabilities might be more reliable than mutant IDH inhibition as they remain disrupted even at later stages of tumor development.

#### 2.2.1. Lipid Metabolism

IDH1 and IDH2 enzymes use NADP^+^ to convert isocitrate to α-KG and produce NADPH, which is important for redox homeostasis and lipogenesis [5,57,58]. However, by mutations in R132 or R172, respectively, IDH1 or IDH2 acquire neomorphic activities to produce D-2-HG from α-KG via consumption of NADPH. Badur et al. (2018) found that increased NADPH consumption inhibits de novo lipogenesis and makes *IDH1*-mutant cells more dependent on exogenous lipid sources for proliferation [59]. They showed that lipid removal from the culture medium significantly decreased the growth of *IDH1*-mutant cells. Therefore, figuring out the in vivo lipid uptake pathway and targeting this mechanism may be a promising strategy for *IDH*-mutant tumors. 

In a recent study, using organelle lipidomics and Raman spectroscopy, it was shown that membrane integrity of especially the ER and Golgi apparatus are disrupted in *IDH1*-mutant glioma cells because of dysregulated lipid metabolism [60]. Besides, it was found that sphingomyelin and monounsaturated fatty acids are accumulated in these organelles in *IDH1*-mutant cells. Based on this finding, the same group recently demonstrated that boosting these pathways via the addition of *N*,*N*-dimethylsphingosine (NDMS) and sphingosine C17 induced an apoptotic response in *IDH1*-mutant glioma cells [61].

#### 2.2.2. Amino Acid Metabolism (Glutamate/Glutamine/Glucose)

Glutamine and glutamate are two five-carbon amino acids that play fundamental roles in cell metabolism as major carbon and nitrogen sources. Glutamine itself is an important precursor for the biosynthesis of proteins, purines, pyrimidines, and amine sugars [62]. However, most of the glutamine is converted to glutamate by the glutaminase (GLS) enzyme to be used in many different metabolic reactions. Glutamate can be used for the synthesis of glutathione, which is an important antioxidant, or converted to α-KG (2-OG) either by glutamate dehydrogenases (GLUDs) or transaminases [63]; α-KG may enter the TCA cycle for energy production or may be used for lipid biosynthesis through reductive carboxylation. Conversely, glutamate can be converted back to glutamine by the glutamine synthetase (GS).

In *IDH*-mutant gliomas, glutamine metabolism has a major role as it has been shown to be altered in many studies [64,65]. As *IDH* mutations are almost always found to be heterozygous, mutant IDH needs the wildtype IDH enzyme to produce α-KG, which is a substrate for the 2-HG production. However, Seltzer et al. (2010) showed that a glutaminase-mediated alternative way of α-KG production is also crucial for *IDH1*-mutant glioma cells probably because of insufficient production from a single allele [66]. Therefore, inhibition of glutaminase slowed down the growth of *IDH1*-mutant glioma cells. Inhibition of the glutamate-to-α-KG conversion also decreased the growth, but only under glucose-deprived conditions [66]. After a few years, Chen et al. (2014) found that expression of GLUD1 and GLUD2, two enzymes converting glutamate to α-KG, increased in *IDH1*-mutant gliomas [67]. They observed that ectopic overexpression of *IDH1^R132H^* inhibited the growth of murine glioma progenitor cells and led to the failure of tumor growth in vivo. However, overexpression of wildtype *IDH1* or *GLUD2* together with *IDH1^R132H^* rescued its growth inhibitory effects in vitro and in vivo [67]. Interestingly, GLUD2 is an enzyme specifically expressed in the hominoid brain and is thought to be evolved from highly conserved GLUD1 during prefrontal cortex evolution [68]. Given that glutamate is an important neurotransmitter found in high levels in the prefrontal cortex, amino acid changes in GLUD2 are thought to ensure efficiency in the acidic microenvironment of astrocytes upon high glutamate intake [69]. As *IDH*-mutant gliomas occur in the prefrontal cortex as well [29], *GLUD2* upregulation provides degradation of the large amount of glutamate to produce α-KG and for biosynthesis of other vital molecules, which is critical for the growth of IDH-mutant cells [67].

On the other hand, branched-chain amino acid (BCAA) transaminases (BCATs), which provide an alternative way of glutamate synthesis, have also been found to be inhibited by 2-HG [70]. Therefore, *IDH*-mutant glioma cells are thought to be more dependent on GLS, whose inhibition leads to glutamate deficiency [70]. As glutamate is a precursor of glutathione (GSH), which is an important antioxidant, GLS inhibition has been shown to sensitize *IDH*-mutant cells to oxidative stress or irradiation [70].

#### 2.2.3. NAD^+^ Metabolism

NAD^+^ is a critical cofactor used in many important metabolic pathways in cells. Upon metabolic profiling of innate *IDH1*-mutant GBM cells, Tateishi et al. (2015) observed increases in the NAD^+^ level with IDH1i treatment and decreases in the NAD^+^ level compared to *IDH1*-wildtype cells because of methylation of the *NAPRT1* promoter [45]. Accordingly, the authors discovered that *IDH1*-mutant cells are more sensitive to NAMPT inhibition, which is responsible for the salvage pathway of NAD^+^ synthesis [45]. In a later study, it was shown that combining NAMPT inhibitors with a DNA-alkylating agent, temozolomide, increased the cytotoxic effects on *IDH1*-mutant cells by increasing NAD^+^ consumption as they are used in base excision repair (BER) upon DNA damage [71]. Similarly, activation of NAD^+^-consuming SIRT1 enzymes by small molecules inhibits the growth of *IDH1*-mutant cells and increases sensitivity to NAMPT inhibition [72]. Another interesting approach is increasing the cytotoxic effect of temozolomide by sequestering NAD^+^ via inhibition of poly(ADP-ribose) glycohydrolase (PARG), which is responsible for NAD^+^ release by the breakdown of PAR chains [73].

As NAD^+^ is required for poly(ADP-ribose) polymerase (PARP)-mediated DNA repair, *IDH1*-mutant cells were thought to have an already impaired PARP-mediated DNA repair because of their decreased NAD^+^ level. Based on that, PARP inhibition has been shown to induce temozolomide cytotoxicity on *IDH1*-mutant glioma cells [74]. However, later studies indicated that sensitizing effects of PARP inhibition are mostly independent of NAD^+^ levels and are rather related with 2-HG-induced homologous recombination (HR) defects [41,73].

#### 2.2.4. Mitochondrial Metabolism and Oxidative Stress

There are many studies demonstrating the effects of *IDH* mutations on mitochondrial metabolism and oxidative stress. Grassian et al. (2014) showed that *IDH*-mutant tumors adapted to use oxidative phosphorylation more than their wildtype counterparts [33]. This made them more dependent on mitochondrial functions and susceptible to inducers of mitochondrial stress or inhibitors of electron transport chain (ETC) enzymes. Another study indicated increased sensitivity of *IDH*-mutant cells to Bcl-2 inhibitors because of the low mitochondrial threshold for apoptosis by 2-HG-mediated inhibition of the cytochrome c oxidase (COX) enzyme [75]. Oxidative pentose phosphate pathway (oxPPP) activity is also increased in *IDH*-mutant tumors to compensate for the decreased NADPH level [59,76]. However, even this increase is not sufficient for all NADPH-mediated reactions. NADPH is known to reduce oxidative stress by neutralizing reactive oxygen species (ROS) through glutathione and thioredoxin systems [77]. Overall, oxidative stress in *IDH*-mutant cells is elevated, which may be one of the reasons for higher sensitivity to radiation therapy [76].

#### 2.2.5. Mammalian Target of Rapamycin (mTOR) Signaling

The mammalian target of rapamycin (mTOR) signaling pathway is one of the major pathways playing role in cellular growth. It takes intracellular and nutritional signals and promotes anabolic reactions, such as protein and lipid synthesis. Several groups observed alterations in mTOR signaling in *IDH*-mutant cells [78,79,80,81]. Fu et al. (2015) found that 2-HG inhibits the ATP synthase (complex V in ETC) and therefore reduces mitochondrial respiration [78]. The decrease in the ATP level leads to the inhibition of mTOR signaling upon both less direct ATP sensing by mTOR, and more AMP sensing by AMPK. This metabolic defect renders *IDH*-mutant cells more sensitive to glucose deprivation. Based on that, fasting and ketogenic diets have been suggested to be beneficial for *IDH*-mutant glioma patients. Similarly, Karpel-Massler et al. (2017) observed a reduction in ATP levels in *IDH*-mutant glioma cells and mTOR inhibition upon AMPK activation [79]. They aimed to boost mitochondrial stress via Bcl-xL inhibitors and showed that *IDH*-mutant cells were more sensitive to Bcl-xL inhibition.

On the other hand, Carbonneau et al. (2016) [80] and Batsios et al. (2019) [81] offered mTOR inhibition as a potential treatment approach. According to the former study, 2-HG inhibits KDM4A, which is responsible for DEPTOR stability; and DEPTOR degradation induces mTORC1/2 signaling [80]. Here, the authors overexpressed mutant *IDH1*/2 in nonmalignant astrocytes and fibroblasts to mimic initial tumorigenesis conditions. Nonetheless, it is known that tertiary mutations in *IDH*-mutant glioma cells activate more aggressive pathways which may repress previous phenotypes [82]. In the latter study, Batsios et al. used a dual PI3K/mTOR inhibitor XL765 and observed growth inhibition in mutant *IDH1* overexpressing normal human astrocytes (NHA) and U87 GBM cells [81]. Interestingly, the authors did not present evidence for the wildtype counterparts. Therefore, it is difficult to say growth inhibition is specific to *IDH*-mutant cells as U87 cells are known to be *PTEN*-null and the mTOR pathway is already active in these cells [83,84].

#### 2.2.6. ER Stress

It has been shown that 2-OG-dependent dioxygenases are inhibited by 2-HG, the product of mutant IDH enzymes [12]. One of these dioxygenases is collagen prolyl-4-hydroxylase (C-P4H). Sasaki et al. found that inhibition of C-P4H by 2-HG impairs collagen maturation and induces an ER stress response because of the accumulation of immature collagens in the ER [85]. Interestingly, *IDH1*-mutant glioma cells were later shown to activate autophagy of the ER (ER-phagy) to survive the 2-HG-mediated ER stress [86]. Based on this observation, inhibition of autophagy via chloroquine (CQ) or bafilomycin A1 (BAF) was shown to be effective in *IDH1*-mutant cells and offered as a potential treatment approach [86]. A recent study also showed that *IDH1*-mutant cells are more sensitive to ER stress-induced apoptosis as miR-183 upregulation in these cells inhibits the antiapoptotic semaphorin 3E [87]. As shown in many other studies of metabolic deficiencies, mutant IDH inhibitor AGI-5198 reversed the stressed phenotype. All these studies indicate an increase in the basal ER stress level in *IDH*-mutant cells, which can be potentiated by pharmacological agents to induce stress-mediated cell death as a promising therapeutic option.

#### 2.2.7. Hypoxia

As the prolyl hydroxylase domain-containing (PHD) enzymes are also 2-OG-dependent (*12*, *13*), they can be inhibited in *IDH*-mutant cells due to the overall decreased levels of 2-OG [88]. Normally, PHD enzymes require 2-OG to hydroxylate specific proline residues of hypoxia-inducible factor 1 (HIF-1α), a major transcription factor responsible for cellular survival and proliferation under hypoxic conditions. This leads to the ubiquitylation and degradation of HIF-1α. Therefore, 2-HG generation and 2-OG depletion indirectly provide the stabilization of HIF-1α, leading to an increase in HIF-1α target genes, such as the vascular endothelial growth factor (VEGF), in *IDH*-mutant gliomas [89]. Based on these studies, inhibition of HIF-1α may be a good strategy to suppress the growth of *IDH*-mutant gliomas. There are many studies where the expression of HIF-1α was inhibited in various ways. These demonstrated inhibition of the growth of gliomas in vitro and in vivo [90,91], decreased glioma cell migration and invasiveness under hypoxic conditions [92], increased efficiency of chemotherapeutic drugs [93], and more sensitivity to radiation therapy [94]. 

Along these lines, it was later shown that D-2-HG, but not L-2-HG, induces the activity of HIF prolyl hydroxylases (EGLN) more than 2-OG itself, resulting in increased HIF-1α degradation [95]. Based on these results, EGLN inhibition was offered as a potential treatment option. Even though this was surprising initially, these results were more consistent with the clinical outcomes, such as a less aggressive phenotype of *IDH*-mutant gliomas [96]. Considering these studies together, *IDH* mutations may cause conflicting changes in both the stability and the degradation of HIF-1α. Induction of L-2-HG production under hypoxia adds to the complexity of the targeting of *IDH*-mutant gliomas [97]. Therefore, the role of hypoxia in the genesis and progression of *IDH*-mutant gliomas is very debatable and further work is needed to dissect out the function of hypoxic regulation in these tumors. Considering mostly oncogenic potentials of HIF-1α, drugs mimicking 2-OG or molecularly targeting HIF-1α still seems like a potent therapeutic approach.

### 2.3. Targeting the DNA Damage Pathway

#### 2.3.1. Conventional Therapies (Temozolomide and Irradiation)

Many studies have shown that *IDH*-mutant gliomas respond better to standard therapy methods such as temozolomide [98,99], irradiation [100,101], and their combination [102,103]. The promoter methylation status of *MGMT*, a DNA repair enzyme involved in the direct repair pathway, is associated with better chemo/radiotherapy response and serves as a prognostic marker in gliomas. Indeed, *MGMT* promoter hypermethylation has been shown to be positively correlated with *IDH1* mutation [104]. However, there are patients with tumors bearing an *IDH* mutation but not *MGMT* promoter methylation. Therefore, the exact mechanisms behind the better response of *IDH*-mutant tumors to conventional therapies are elusive. Despite better response to standard therapies, most of the lower-grade *IDH*-mutant gliomas still progress to high-grade levels [105].

#### 2.3.2. PARP-mediated DNA Repair

Two independent studies in 2017 proposed that targeting PARP-mediated DNA repair could be a promising strategy for *IDH*-mutant gliomas, even if each claimed a different molecular mechanism responsible for this finding [104]. Both groups showed that the NAD^+^ level was slightly decreased in IDH-mutant cells, in line with previous reports [45]. According to Lu et al. (2017), the lower NAD^+^ level leads to more DNA damage upon temozolomide treatment because of the impaired PARP-mediated repair, which uses NAD^+^ as a substrate; and this can be boosted by PARP inhibitors [74]. On the other hand, Sulkowski et al. (2017) indicated 2-HG-mediated inhibition of KDM4A and KDM4B enzymes, which are thought to be responsible for double-strand break (DSB) repair via homologous recombination (HR) [41]. The authors claimed that inhibition of HR confers a “BRCAness” phenotype and renders *IDH*-mutant glioma cells sensitive to PARP inhibitors. This phenotype can also be reversed by mutant IDH1 inhibitors, AGI-5198, AG-120, and IDH1-C227 [41]. Similarly, a recent study showed that PARP inhibition together with radiation-induced DNA damage is highly effective in both in vitro and in vivo models of *IDH*-mutant gliomas [106]. Based on these promising studies, PARP inhibitors are being tested in ongoing clinical trials individually (Table 3) or in combination with temozolomide or immune checkpoint inhibitor durvalumab (Table 2).

### 2.4. Immunotherapy

Even though the mutational load is relatively low in GBMs [108], heterogeneity is still one of the most important causes of therapy resistance and recurrence [109]. Since *IDH* mutations are early events in gliomagenesis and are homogenously found at specific codons in all glioma cells, they are suggested as a potential target for an immunotherapeutic approach [110]. On the other hand, multiple studies have indicated that the genes responsible for the production of immune cell-attracting chemokines are suppressed in *IDH*-mutant gliomas [111,112,113,114]. Based on these studies, a combinatorial approach successfully demonstrated that the combination of mutant IDH inhibitors with vaccination therapy or immune checkpoint inhibitors overcomes the mutant IDH-dependent immune evasion [111,114]. In a recent study, the combination of a mutant IDH inhibitor with temozolomide/IR and an immune checkpoint inhibitor enhanced survival. This combinatorial approach was more efficient than each individual treatment in an *IDH1^R132H^*-bearing mouse glioma model [115].

#### 2.4.1. Peptide Vaccines

It has been shown that *IDH1^R132H^* mutants have an immunogenic epitope suitable for mutation-specific vaccination [110]. The peptides surrounding this mutated region belong to major histocompatibility complexes (MHC) class II and promote the IDH1^R132H^-specific CD4+ T helper 1 (TH1) responses in patients with mutant IDH. Considering this significant finding, vaccines targeting this specific epitope were developed and found highly effective in an intracranial murine glioma model [116]. NOA-16, an IDH1^R132H^ peptide vaccine, is a first-in-human, multicenter, phase I clinical trial (Table 3). The first report validated the safety and therapeutic efficacy of NOA-16 in the patients newly diagnosed with malignant astrocytoma harboring the *IDH1* mutation (NCT02454634) [117]. In a recent report of this trial, vaccine-induced immune responses were seen in 93.3% of the patients [107]. The authors also showed the presence of tumor-infiltrating CD40LG+ and CXCL13+ T helper cell clusters dominated by a single IDH1^R132H^-reactive T cell receptor in a patient with pseudoprogression. Considering the disease progression in patients who did not develop a vaccine-induced immune response, this strategy seems promising in terms of efficacy as well. There is one more IDH1 peptide vaccine, PEPIDH1M, for recurrent grade II gliomas (RESIST) the safety of which is now being tested in combination with the standard chemotherapy, temozolomide (NCT02193347). Another clinical trial for an IDH1^R132H^-dendritic cell vaccine was launched to test the safety and effectiveness in patients with *IDH*-mutant gliomas (NCT02771301). The results of these trials have not been published yet.

#### 2.4.2. Immune Checkpoint Inhibitors

Immune checkpoint inhibitors such as avelumab comprise other promising agents to suppress the immune evasion of tumor cells [42]. They basically work by blocking the interaction between cytotoxic T lymphocytes and immune checkpoint ligands, resulting in the abolition of the ligands’ suppressive effects on lymphocytes. There is a phase II clinical trial in which avelumab associated with hypofractionated radiation therapy is investigated in patients with *IDH*-mutant glioblastomas (Table 2). It was completed, but its result has not been reported yet (NCT02968940). In another clinical study called AMPLIFY-NEOVAC (AMPLIFYing NEOepitope-specific VACcine Responses in Progressive Diffuse Glioma), avelumab associated with the IDH1^R132H^ peptide vaccine is being tested for safety and tolerability of the combination (NCT03893903). There are other ongoing clinical trials of immune checkpoint inhibitors nivolumab and durvalumab in patients with IDH-mutant gliomas (NCT03991832, NCT03557359, NCT03718767, NCT03925246) (Table 2 and Table 3).

#### 2.4.3. CAR T Cell Therapy

Some specific antigens may be found on the surface of glioma cells, for example, EGFR variant III (EGFRvIII). They can be targeted by the genetically modified chimeric antigen receptor (CAR) T cells or oncolytic viral therapy, in which viruses are genetically engineered in order to selectively infect and replicate in tumor cells, therefore resulting in not only cellular lysis, but also the activation of immunogenic cell death pathways [118,119]. Even if EGFRvIII is mostly associated with *IDH*-wildtype gliomas, there are some cases in which *IDH* mutation and *EGFRvIII* are found together [120,121]. Alternatively, PDGFRA, which is known to be upregulated in IDH-mutant gliomas [122], has recently been shown to be targeted by CAR T cell therapy in other cancer types [123]. Therefore, targeting neoantigens with CAR T cell therapy might be an interesting treatment option for IDH-mutant gliomas.

### 2.5. Epigenetic Approaches

#### 2.5.1. DNA Demethylation

Considering the well-established hypermethylated phenotype in *IDH*-mutant tumors, DNA demethylation agents were among the first agents tested as a therapeutic approach. In 2013, two companion papers indicated that 5-azacytidine and decitabine, which are well-known DNA methyltransferase inhibitors (DNMTi), induced differentiation of *IDH1*-mutant glioma cells and inhibited tumor growth in xenograft models [39,124].

In 2017, the group that published one of the papers in 2013 demonstrated that the effect of 5-azacytidine can be enhanced when combined with temozolomide [125]. As a single agent, 5-azacytidine had similar effects on *IDH1*-wildtype and -mutant glioma cells in vitro and slightly reduced *IDH1*-mutant tumor growth in vivo. However, the combination with temozolomide further decreased tumor volume and increased survival in both subcutaneous and orthotopic glioma models. They also tested the mutant IDH1 inhibitor, AGI-5198, and found that it has no effect on the proliferation of *IDH1*-mutant glioma cells neither individually nor with 5-azacytidine.

Based on these results, there are ongoing clinical trials testing 5-azacytidine individually or in combination with mutant IDH inhibitors (Table 2). Another clinical trial testing ASTX727, which is a fixed-dose combination of cedazuridine and decitabine, is also being conducted in *IDH*-mutant glioma patients (NCT03922555). Cedazuridine is an inhibitor of cytidine deaminase (CDA) and has been shown to inhibit CDAs in the gut and the liver, allowing to achieve high plasma concentration of orally delivered decitabine [126,127].

#### 2.5.2. BET Inhibitors

Bai et al. (2016) identified activation of aggressive oncogenic pathways such as the MYC or RTK-RAS-PI3K signaling pathways as well as important epigenetic pathways evident in the progression of *IDH1*-mutant gliomas [128]. Based on these alterations and the previous studies showing the efficacy of bromodomain and extraterminal motif (BET) inhibitors for primary GBMs, the authors tested BET inhibitors on *IDH1*-mutant glioma cells. They observed high sensitivity of patient-derived *IDH1*-mutant glioma cells with submicromolar IC50 values, which are several orders of magnitude lower than IC50 of temozolomide [128]. Therefore, BET inhibitors also offer a clinical potential in *IDH*-mutant gliomas.

#### 2.5.3. Combination Treatments

*IDH1*-mutant tumors are known to have a distinct epigenetic profile due to mutant *IDH1*-dependent chromatin modifications [47]. Prolonged presence of mutant IDH1 renders most of these modifications irreversible, which may have critical importance for tumor progression. Based on this epigenetic reprogramming, we recently conducted chemical screening in *IDH1*-mutant GBM cells, including inhibitors of epigenetic enzymes [36]. We found that *IDH1*-mutant GBM cells are sensitive to different epigenetic enzyme inhibitors such as DNMT inhibitor 5-azacitidine, HMT inhibitor chaetocin, KDM inhibitor GSK-J4, and HDAC inhibitor belinostat. Moreover, combined inhibition of KDM6A/B and HDACs was markedly more effective than individual treatments of *IDH1*-mutant gliomas. These results may indicate that *IDH1*-mutant tumors are dependent on their distinct epigenome and exploiting this distinct phenotype via chemical inhibitors may lead to the development of successful therapeutic approaches.

## 3. Conclusions

*IDH* mutations are highly frequent in lower-grade gliomas and secondary GBMs. Although they have been shown to be less aggressive than *IDH*-wildtype gliomas, there is still no approved therapy for *IDH*-mutant gliomas. Considering that they are among the driver mutations in gliomagenesis, many mutant IDH inhibitors are being developed to reverse this phenotype. Even if some of these inhibitors were approved for AML treatment, their efficacy in gliomas has been shown to be dismal. There are still ongoing clinical trials in which mutant IDH inhibitors are being used individually or in combination with other treatment options. On the other hand, mutant IDH can induce unique dependencies and weaknesses in tumor cells, which can be exploited as an alternative therapeutic approach. Targeting metabolic deficiencies or DNA damage pathways has already been shown to be effective in preclinical studies. Based on the highly specific and homogenous nature of IDH mutations, immunotherapeutic options targeting IDH1^R132H^ have also been offered as an alternative treatment method. Lastly, the distinct epigenetic profile of *IDH*-mutant gliomas makes it possible to develop potential therapeutic approaches using epigenetic enzyme inhibitors. Overall, there are many alternative approaches shown to be effective in preclinical studies for *IDH*-mutant gliomas, which increase the chance of having an approved treatment upon completion of clinical trials.

## Figures and Tables

**Table 1:** Clinical trials with mIDHi in IDH-mutant gliomas.

Mutant IDH Inhibitors					
ClinicalTrials.gov Identifier	Drug	Mechanism of Action	Phase	Status	Announced Results
NCT02073994	Ivosidenib (AG-120)	mIDH1 inhibitor	Phase I	Active, not recruiting	Favorable safety profile. Reduction in volume and growth rate of only non-enhancing gliomas [50]
NCT03030066	DS-1001b	mIDH1 inhibitor	Phase I	Active, not recruiting	Favorable safety profile under 100 mg/day. One partial response and one minor response in 14 patients [51]
NCT02381886	IDH305	mIDH1 inhibitor	Phase I	Active, not recruiting	Favorable safety profile, therapeutic responses were announced only for AML [52]
NCT02746081	BAY1436032	mIDH1 inhibitor	Phase I	Active, not recruiting	
NCT04458272	DS-1001b	mIDH1 inhibitor	Phase II	Active, not recruiting	
NCT02977689	IDH305	mIDH1 inhibitor	Phase II	Withdrawn	Novartis paused all study start-up activities due to the safety evaluation of the *IDH305* compound
NCT02987010	IDH305	mIDH1 inhibitor	Phase II	Withdrawn	The sponsor did not want to move forward with the protocol; the study was never opened
NCT04521686	LY3410738	mIDH1 inhibitor	Phase I	Recruiting	
NCT04195555	Ivosidenib (AG-120)	mIDH1 inhibitor	Phase II	Recruiting	
NCT02273739	Enasidenib (AG-221)	mIDH2 inhibitor	Phase I/II	Completed	Closed earlier than planned. High ratio of adverse events
NCT02481154	Vorasidenib (AG-881)	mIDH1/2 inhibitor	Phase I	Active, not recruiting	Favorable safety profile under 100 mg/day. Efficacy data not announced yet
NCT04762602	HMPL-306	m*IDH1/2* inhibitor	Phase I	Recruiting	
NCT04164901	Vorasidenib (AG-881)	m*IDH1/2* inhibitor	Phase III	Recruiting	
NCT03343197	Ivosidenib (AG-120) or vorasidenib (AG-881)	mIDH1 or m*IDH1/2* inhibitor	Phase I	Active, not recruiting	

**Table 2:** Clinical trials with combinatorial approaches in IDH-mutant gliomas.

Combinatorial Approaches					
ClinicalTrials.gov Identifier	Drug	Mechanism of Action	Phase	Status	Announced Results
NCT03893903	IDH1^R132H^ peptide vaccine and avelumab	Combination of an IDH1^R132H^-specific vaccine and an immune checkpoint inhibitor	Phase I	Recruiting	
NCT03749187	BGB-290 and temozolomide	Combination of the PARP1/2 inhibitor and an alkylating agent	Phase I	Recruiting	
NCT02193347	IDH1 peptide vaccine and temozolomide	Combination of a PEPIDH1M vaccine and an alkylating agent	Phase I	Active, not recruiting	
NCT04056910	Ivosidenib (AG-120) with nivolumab	Combination of an mIDH1 inhibitor and an immune checkpoint inhibitor	Phase II	Recruiting	
NCT03991832	Olaparib and durvalumab	Combination of a PARP inhibitor and an immune checkpoint inhibitor	Phase II	Recruiting	
NCT03180502	Proton beam or intensity-modulated radiation therapy and temozolomide	Combination of radiation and an alkylating agent	Phase II	Recruiting	
NCT02968940	Avelumab and hypofractionated radiation therapy	Combination of an immune checkpoint inhibitor and radiation	Phase II	Completed	
NCT03914742	BGB-290 and temozolomide	Combination of a PARP1/2 inhibitor and an alkylating agent	Phase I/II	Recruiting	
NCT03684811	Olutasidenib (FT-2102) and azacitidine	Single-agent treatment with an mIDH1 inhibitor and a combination with a DNA methyltransferase 1 inhibitor	Phase I/II	Active, not recruiting	Favorable safety profile, low efficacy with a single agent, evaluation of the combination is ongoing [53]
NCT02496741	Metformin and chloroquine	Combination of antidiabetic and antimalarial agents	Phase I/II	Completed	
NCT03528642	CB-839 with radiation therapy and temozolomide	Combination of radiation and an alkylating agent with a glutaminase inhibitor	Phase I	Recruiting	
NCT00626990	Radiation and temozolomide	Combination of radiation and an alkylating agent	Phase III	Active, not recruiting	Increased overall survival with adjuvant TMZ in IDH-mutant gliomas [54]

**Table 3:** Clinical trials with targeted therapies in IDH-mutant gliomas.

**DNA Methyltransferase (DNMT) Inhibitors**					
**ClinicalTrials.gov Identifier**	**Drug**	**Mechanism of Action**	**Phase**	**Status**	**Announced Results**
NCT03922555	ASTX727	DNMT and cytidine deaminase inhibitor	Phase I	Recruiting	
NCT03666559	Azacitidine	DNMT1 inhibitor	Phase II	Recruiting	
**Poly ADP Ribose Polymerase (PARP) Inhibitors**					
**ClinicalTrials.gov Identifier**	**Drug**	**Mechanism of Action**	**Phase**	**Status**	
NCT03212274	Olaparib (AZD2281)	PARP1/2 inhibitor	Phase II	Recruiting	
NCT03561870	Olaparib	PARP1/2 inhibitor	Phase II	Active, not recruiting	
**Immunotherapy**					
**ClinicalTrials.gov Identifier**	**Drug**	**Mechanism of Action**	**Phase**	**Status**	
NCT02454634	IDH1 peptide vaccine (NOA-16)	IDH1 peptide vaccine	Phase I	Completed	Favorable safety profile, high percentage of IDH peptide-specific immune response [107]
NCT02771301	*IDH1^R132H^*-DC vaccine	IDH1^R132H^-dendritic cell vaccine	Phase I	Unknown	
NCT03557359	Nivolumab	Immune checkpoint inhibitor	Phase II	Recruiting	
NCT03718767	Nivolumab	Immune checkpoint inhibitor	Phase II	Recruiting	
NCT03925246	Nivolumab	Immune checkpoint inhibitor	Phase II	Active, not recruiting	

## References

[1] Ostrom Q.T., Gittleman H., Xu J., Kromer C., Wolinsky Y., Kruchko C., Barnholtz-Sloan J.S. (2016). CBTRUS Statistical Report: Primary Brain and Other Central Nervous System Tumors Diagnosed in the United States in 2009–2013. Neuro-Oncology.

[2] Louis D.N., Perry A., Reifenberger G., von Deimling A., Figarella-Branger D., Cavenee W.K., Ohgaki H., Wiestler O.D., Kleihues P., Ellison D.W. (2016). The 2016 World Health Organization Classification of Tumors of the Central Nervous System: A summary. Acta Neuropathol..

[3] Bowman R.L., Wang Q., Carro A., Verhaak R.G.W., Squatrito M. (2017). GlioVis data portal for visualization and analysis of brain tumor expression datasets. Neuro-Oncology.

[4] Hurley J.H., Dean A.M., Koshland D.E., Stroud R.M. (1991). Catalytic Mechanism of NADP+-Dependent Isocitrate Dehydrogenase: Implications from the Structures of Magnesium-Isocitrate and NADP + Complexes. Biochemistry.

[5] Lee S.M., Koh H.J., Park D.C., Song B.J., Huh T.L., Park J.W. (2002). Cytosolic NADP+-dependent isocitrate dehydrogenase status modulates oxidative damage to cells. Free Radic. Biol. Med..

[6] Losman J.-A., Kaelin W.G. (2013). What a difference a hydroxyl makes: Mutant *IDH*, (R)-2-hydroxyglutarate, and cancer. Genes Dev..

[7] Ramachandran N., Colman R.F. (1980). Chemical characterization of distinct subunits of pig heart DPN-specific isocitrate dehy-drogenase. J. Biol. Chem..

[8] Xu X., Zhao J., Xu Z., Peng B., Huang Q., Arnold E., Ding J. (2004). Structures of human cytosolic NADP-dependent isocitrate dehydrogenase reveal a novel self-regulatory mechanism of activity. J. Biol. Chem..

[9] Gabriel J.L., Zervos P.R., Plaut G.W.E. (1986). Activity of purified NAD-specific isocitrate dehydrogenase at modulator and sub-strate concentrations approximating conditions in mitochondria. Metabolism.

[10] Hartmann C., Meyer J., Balss J., Capper D., Mueller W., Christians A., Felsberg J., Wolter M., Mawrin C., Wick W. (2009). Type and frequency of *IDH1* and *IDH2* mutations are related to astrocytic and oligodendroglial differentiation and age: A study of 1,010 diffuse gliomas. Acta Neuropathol..

[11] Ye D., Xiong Y., Guan K.-L. (2012). The mechanisms of *IDH* mutations in tumorigenesis. Cell Res..

[12] Xu W., Yang H., Liu Y., Yang Y., Wang P., Kim S.-H., Ito S., Yang C., Wang P., Xiao M.-T. (2011). Oncometabolite 2-hydroxyglutarate is a competitive inhibitor of α-ketoglutarate-dependent dioxygenases. Cancer Cell.

[13] Chowdhury R., Yeoh K.K., Tian Y.-M., Hillringhaus L., Bagg E.A., Rose N.R., Leung I.K.H., Li X.S., Woon E.C.Y., Yang M. (2011). The oncometabolite 2-hydroxyglutarate inhibits histone lysine demethylases. EMBO Rep..

[14] Turcan S., Rohle D., Goenka A., Walsh L.A., Fang F., Yilmaz E., Campos C., Fabius A.W.M.M., Lu C., Ward P.S. (2012). *IDH1* mutation is sufficient to establish the glioma hypermethylator phenotype. Nature.

[15] Lu C., Ward P.S., Kapoor G.S., Rohle D., Turcan S., Abdel-Wahab O., Edwards C.R., Khanin R., Figueroa M.E., Melnick A. (2012). *IDH* mutation impairs histone demethylation and results in a block to cell differentiation. Nature.

[16] Noorani I. (2019). Genetically engineered mouse models of gliomas: Technological developments for translational discoveries. Cancers.

[17] Philip B., Yu D.X., Silvis M.R., Shin C.H., Robinson J.P., Robinson G.L., Welker A.E., Angel S.N., Tripp S.R., Sonnen J.A. (2018). Mutant *IDH1* Promotes Glioma Formation In Vivo. Cell Rep..

[18] Bettegowda C., Agrawal N., Jiao Y., Sausen M., Wood L.D., Hruban R.H., Rodriguez F.J., Cahill D.P., McLendon R., Riggins G. (2011). Mutations in CIC and FUBP1 Contribute to Human Oligodendroglioma. Science.

[19] Yip S., Butterfield Y.S., Morozova O., Chittaranjan S., Blough M.D., An J., Birol I., Chesnelong C., Chiu R., Chuah E. (2012). Concurrent CIC mutations, *IDH* mutations, and 1p/19q loss distinguish oligodendrogliomas from other cancers. J. Pathol..

[20] The Cancer Genome Atlas Research Network (2015). Comprehensive, Integrative Genomic Analysis of Diffuse Lower-Grade Gliomas. N. Engl. J. Med..

[21] Suzuki H., Aoki K., Chiba K., Sato Y., Shiozawa Y., Shiraishi Y., Shimamura T., Niida A., Motomura K., Ohka F. (2015). Mutational landscape and clonal architecture in grade II and III gliomas. Nat. Genet..

[22] Metellus P., Coulibaly B., Colin C., de Paula A.M., Vasiljevic A., Taieb D., Barlier A., Boisselier B., Mokhtari K., Wang X.W. (2010). Absence of *IDH* mutation identifies a novel radiologic and molecular subtype of WHO grade II gliomas with dismal prognosis. Acta Neuropathol..

[23] Noorani I., de la Rosa J., Choi Y.H., Strong A., Ponstingl H., Vijayabaskar M.S., Lee J., Lee E., Richard-Londt A., Frie-drich M. (2020). PiggyBac mutagenesis and exome sequencing identify genetic driver landscapes and potential therapeutic targets of EGFR-mutant gliomas. Genome Biol..

[24] Nobusawa S., Watanabe T., Kleihues P., Ohgaki H. (2009). *IDH1* mutations as molecular signature and predictive factor of sec-ondary glioblastomas. Clin. Cancer Res..

[25] Ohgaki H., Dessen P., Jourde B., Horstmann S., Nishikawa T., di Patre P.L., Burkhard C., Schüler D., Probst-Hensch N.M., Maiorka P.C. (2004). Genetic pathways to glioblastoma: A population-based study. Cancer Res..

[26] Bender S., Tang Y., Lindroth A.M., Hovestadt V., Jones D.T.W., Kool M., Zapatka M., Northcott P.A., Sturm D., Wang W. (2013). Reduced H3K27me3 and DNA Hypomethylation Are Major Drivers of Gene Expression in K27M Mutant Pediatric High-Grade Gliomas. Cancer Cell.

[27] Johnson B.E., Mazor T., Hong C., Barnes M., Aihara K., McLean C.Y., Fouse S.D., Yamamoto S., Ueda H., Tatsuno K. (2014). Mutational Analysis Reveals the Origin and Therapy-Driven Evolution of Recurrent Glioma. Science.

[28] Watanabe T., Nobusawa S., Kleihues P., Ohgaki H. (2009). *IDH1* mutations are early events in the development of astrocytomas and oligodendrogliomas. Am. J. Pathol..

[29] Lai A., Kharbanda S., Pope W.B., Tran A., Solis O.E., Peale F., Forrest W.F., Pujara K., Carrillo J.A., Pandita A. (2011). Evidence for sequenced molecular evolution of *IDH1* mutant glioblastoma from a distinct cell of origin. J. Clin. Oncol..

[30] Rohle D., Popovici-Muller J., Palaskas N., Turcan S., Grommes C., Campos C., Tsoi J., Clark O., Oldrini B., Komi-sopoulou E. (2013). An inhibitor of mutant *IDH1* delays growth and promotes differentiation of glioma cells. Science.

[31] Wang F., Travins J., DeLaBarre B., Penard-Lacronique V., Schalm S., Hansen E., Straley K., Kernytsky A., Liu W., Gliser C. (2013). Targeted Inhibition of Mutant *IDH2* in Leukemia Cells Induces Cellular Differentiation. Science.

[32] Okoye-Okafor U.C., Bartholdy B., Cartier J., Gao E.N., Pietrak B., Rendina A.R., Rominger C., Quinn C., Smallwood A., Wiggall K.J. (2015). New *IDH1* mutant inhibitors for treatment of acute myeloid leukemia. Nat. Chem. Biol..

[33] Grassian A.R., Parker S.J., Davidson S.M., Divakaruni A.S., Green C.R., Zhang X., Slocum K.L., Pu M., Lin F., Vickers C. (2014). *IDH1* mutations alter citric acid cycle metabolism and increase dependence on oxidative mitochondrial metabolism. Cancer Res..

[34] Horton J.R., Engstrom A., Zoeller E.L., Liu X., Shanks J.R., Zhang X., Johns M.A., Vertino P.M., Fu H., Cheng X. (2016). Char-acterization of a Linked Jumonji Domain of the KDM5/JARID1 Family of Histone H3 Lysine 4 Demethylases. J. Biol. Chem..

[35] Chesnelong C., Chaumeil M.M., Blough M.D., Al-Najjar M., Stechishin O.D., Chan J.A., Pieper R.O., Ronen S.M., Weiss S., Luchman H.A. (2014). Lactate dehydrogenase A silencing in *IDH* mutant gliomas. Neuro-Oncology.

[36] Kayabolen A., Sahin G.N., Seker-Polat F., Cingoz A., Isik B., Acar S., Wakimoto H., Cahill D.P., Solaroglu I., Cribbs A. (2020). Combined inhibition of KDM6A/B and HDACs exacerbates integrated stress response and mediates therapeutic effects in *IDH1*-mutant glioma. bioRxiv.

[37] Kopinja J., Sevilla R.S., Levitan D., Dai D., Vanko A., Spooner E., Ware C., Forget R., Hu K., Kral A. (2017). A Brain Pen-etrant Mutant *IDH1* Inhibitor Provides in Vivo Survival Benefit. Sci. Rep..

[38] Pusch S., Krausert S., Fischer V., Balss J., Ott M., Schrimpf D., Capper D., Sahm F., Eisel J., Beck A.-C. (2017). Pan-mutant *IDH1* inhibitor BAY 1436032 for effective treatment of *IDH1* mutant astrocytoma in vivo. Acta Neuropathol..

[39] Turcan S., Fabius A.W., Borodovsky A., Pedraza A., Brennan C., Huse J., Viale A., Riggins G.J., Chan T.A., Turcan S. (2013). Efficient induction of differentiation and growth inhibition in *IDH1* mutant glioma cells by the DNMT Inhibitor Decita-bine. Oncotarget.

[40] Molenaar R.J., Botman D., Smits M.A., Hira V.V., van Lith S.A., Stap J., Henneman P., Khurshed M., Lenting K., Mul A.N. (2015). Radioprotection of *IDH1*-mutated cancer cells by the *IDH1*-mutant inhibitor AGI-5198. Cancer Res..

[41] Sulkowski P.L., Corso C.D., Robinson N.D., Scanlon S.E., Purshouse K.R., Bai H., Liu Y., Sundaram R.K., Hegan D.C., Fons N.R. (2017). 2-Hydroxyglutarate produced by neomorphic *IDH* mutations suppresses homologous recombination and induces PARP inhibitor sensitivity. Sci. Transl. Med..

[42] Kim E.S. (2017). Avelumab: First Global Approval. Drugs.

[43] Norsworthy K.J., Luo l., Hsu V., Gudi R., Dorff S.E., Przepiorka D., Deisseroth A., Shen Y.-L., Sheth C.M., Charlab R. (2019). FDA Approval Summary: Ivosidenib for Relapsed or Refractory Acute Myeloid Leukemia with an Isocitrate Dehydrogenase-1 Mutation. Clin. Cancer Res..

[44] Andronesi O.C., Arrillaga-Romany I.C., Ly K.I., Bogner W., Ratai E.M., Reitz K., Iafrate A.J., Dietrich J., Gerstner E.R., Chi A.S. (2018). Pharmacodynamics of mutant-IDH1 inhibitors in glioma patients probed by in vivo 3D MRS imaging of 2-hydroxyglutarate. Nat. Commun..

[45] Tateishi K., Wakimoto H., Iafrate A.J., Tanaka S., Loebel F., Lelic N., Wiederschain D., Bedel O., Deng G., Zhang B. (2015). Extreme Vulnerability of *IDH1* Mutant Cancers to NAD+ Depletion. Cancer Cell.

[46] DiNardo C.D., Stein E.M., de Botton S., Roboz G.J., Altman J.K., Mims A.S., Swords R., Collins R.H., Mannis G.N., Pol-lyea D.A. (2018). Durable remissions with ivosidenib in *IDH1*-mutated relapsed or refractory AML. N. Engl. J. Med..

[47] Turcan S., Makarov V., Taranda J., Wang Y., Fabius A.W.M., Wu W., Zheng Y., El-Amine N., Haddock S., Nanjangud G. (2018). Mutant-IDH1-dependent chromatin state reprogramming, reversibility, and persistence. Nat. Genet..

[48] Johannessen T.-C.A., Mukherjee J., Viswanath P., Ohba S., Ronen S.M., Bjerkvig R., Pieper R.O. (2016). Rapid Conversion of Mutant *IDH1* from Driver to Passenger in a Model of Human Gliomagenesis. Mol. Cancer Res..

[49] Kats L.M., Reschke M., Taulli R., Pozdnyakova O., Burgess K., Bhargava P., Straley K., Karnik R., Meissner A., Small D. (2014). Proto-oncogenic role of mutant *IDH2* in leukemia initiation and maintenance. Cell Stem Cell.

[50] Mellinghoff I.K., Ellingson B.M., Touat M., Maher E., de la Fuente M.I., Holdhoff M., Cote G.M., Burris H., Janku F., Young R.J. (2020). Ivosidenib in Isocitrate Dehydrogenase 1-Mutated Advanced Glioma. J. Clin. Oncol..

[51] Natsume A., Wakabayashi T., Miyakita Y., Narita Y., Mineharu Y., Arakawa Y., Yamasaki F., Sugiyama K., Hata N., Muragaki Y. (2019). Phase I study of a brain penetrant mutant *IDH1* inhibitor DS-1001b in patients with recurrent or progressive *IDH1* mutant gliomas. J. Clin. Oncol..

[52] DiNardo C.D., Schimmer A.D., Yee K.W.L., Hochhaus A., Kraemer A., Carvajal R.D., Janku F., Bedard P., Carpio C., Wick A. (2016). A Phase I Study of *IDH305* in Patients with Advanced Malignancies Including Relapsed/Refractory AML and MDS That Harbor IDH1R132 Mutations. Blood.

[53] de la Fuente M.I., Colman H., Rosenthal M., van Tine B.A., Levaci D., Walbert T., Gan H.K., Vieito M., Milhem M.M., Lipford K. (2020). A phase Ib/II study of olutasidenib in patients with relapsed/refractory *IDH1* mutant gliomas: Safety and efficacy as single agent and in combination with azacitidine. J. Clin. Oncol..

[54] van den Bent M.J., Erridge S., Vogelbaum M.A., Nowak A.K., Sanson M., Brandes A.A., Wick W., Clement P.M., Baurain J.-F., Mason W.P. (2019). Second interim and first molecular analysis of the EORTC randomized phase III intergroup CAT-NON trial on concurrent and adjuvant temozolomide in anaplastic glioma without 1p/19q codeletion. J. Clin. Oncol..

[55] Warburg O. (1956). On the origin of cancer cells. Science.

[56] Zecchini V., Frezza C. (2017). Metabolic synthetic lethality in cancer therapy. Biochim. Biophys. Acta Bioenerg..

[57] Jo S.H., Son M.K., Koh H.J., Lee S.M., Song I.H., Kim Y.O., Lee Y.S., Jeong K.S., Kim W.B., Park J.W. (2001). Control of Mitochondrial Redox Balance and Cellular Defense against Oxidative Damage by Mitochondrial NADP+-dependent Isocitrate Dehydrogenase. J. Biol. Chem..

[58] Koh H.J., Lee S.M., Son B.G., Lee S.H., Ryoo Z.Y., Chang K.T., Park J.W., Park D.C., Song B.J., Veech R.L. (2004). Cytosolic NADP+-dependent isocitrate dehydrogenase plays a key role in lipid metabolism. J. Biol. Chem..

[59] Badur M.G., Muthusamy T., Parker S.J., Ma S., McBrayer S.K., Cordes T., Magana J.H., Guan K.L., Metallo C.M. (2018). Onco-genic R132 *IDH1* Mutations Limit NADPH for De Novo Lipogenesis through (D)2-Hydroxyglutarate Production in Fibro-sarcoma Sells. Cell Rep..

[60] Lita A., Pliss A., Kuzmin A., Yamasaki T., Zhang L., Dowdy T., Burks C., de Val N., Celiku O., Ruiz-Rodado V. (2020). *IDH1* Mutations Induce Organelle Defects Via Dysregulated Phospholipids. bioRxiv.

[61] Dowdy T., Zhang L., Celiku O., Movva S., Lita A., Ruiz-Rodado V., Gilbert M.R., Larion M. (2020). Sphingolipid pathway as a source of vulnerability in IDH1mut glioma. Cancers.

[62] Newsholme P., Procopio J., Lima M.M.R., Pithon-Curi T.C., Curi R. (2003). Glutamine and glutamate?their central role in cell metabolism and function. Cell Biochem. Funct..

[63] Altman B.J., Stine Z.E., Dang C.V. (2016). From Krebs to clinic: Glutamine metabolism to cancer therapy. Nat. Rev. Cancer.

[64] Fack F., Tardito S., Hochart G., Oudin A., Zheng L., Fritah S., Golebiewska A., Nazarov P.V., Bernard A., Hau A. (2017). Altered metabolic landscape in *IDH*-mutant gliomas affects phospholipid, energy, and oxidative stress pathways. EMBO Mol. Med..

[65] Salamanca-Cardona L., Shah H., Poot A.J., Correa F.M., di Gialleonardo V., Lui H., Miloushev V.Z., Granlund K.L., Tee S.S., Cross J.R. (2017). In Vivo Imaging of Glutamine Metabolism to the Oncometabolite 2-Hydroxyglutarate in *IDH1/2* Mu-tant Tumors. Cell Metab..

[66] Seltzer M.J., Bennett B.D., Joshi A.D., Gao P., Thomas A.G., Ferraris D.V., Tsukamoto T., Rojas C.J., Slusher B.S., Rab-inowitz J.D. (2010). Inhibition of glutaminase preferentially slows growth of glioma cells with mutant *IDH1*. Cancer Res..

[67] Chen R., Nishimura M.C., Kharbanda S., Peale F., Deng Y., Daemen A., Forrest W.F., Kwong M., Hedehus M., Hatzi-vassiliou G. (2014). Hominoid-specific enzyme GLUD2 promotes growth of *IDH1* R132H glioma. Proc. Natl. Acad. Sci. USA.

[68] Burki F., Kaessmann H. (2004). Birth and adaptive evolution of a hominoid gene that supports high neurotransmitter flux. Nat. Genet..

[69] Shashidharan P., Plaitakis A. (2014). The discovery of human of GLUD2 glutamate dehydrogenase and its implications for cell function in health and disease. Neurochem. Res..

[70] McBrayer S.K., Mayers J.R., DiNatale G.J., Shi D.D., Khanal J., Chakraborty A.A., Sarosiek K.A., Briggs K.J., Robbins A.K., Sewastianik T. (2018). Transaminase Inhibition by 2-Hydroxyglutarate Impairs Glutamate Biosynthesis and Redox Homeostasis in Glioma. Cell.

[71] Tateishi K., Higuchi F., Miller J.J., Koerner M.V.A., Lelic N., Shankar G.M., Tanaka S., Fisher D.E., Batchelor T.T., Iafrate A.J. (2017). The Alkylating Chemotherapeutic Temozolomide Induces Metabolic Stress in *IDH1* -Mutant Cancers and Poten-tiates NAD + Depletion–Mediated Cytotoxicity. Cancer Res..

[72] Miller J.J., Fink A., Banagis J.A., Nagashima H., Subramanian M., Lee C.K., Melamed L., Tummala S.S., Tateishi K., Wakimoto H. (2020). Sirtuin activation targets *IDH*-mutant tumors. Neuro-Oncology.

[73] Nagashima H., Lee C.K., Tateishi K., Higuchi F., Subramanian M., Rafferty S., Melamed L., Miller J.J., Wakimoto H., Cahill D.P. (2020). Poly(ADP-ribose) Glycohydrolase Inhibition Sequesters NAD+ to Potentiate the Metabolic Lethality of Alkyl-ating Chemotherapy in *IDH*-Mutant Tumor Cells. Cancer Discov..

[74] Lu Y., Kwintkiewicz J., Liu Y., Tech K., Frady L.N., Su Y.-T., Bautista W., Moon S.I., MacDonald J., Ewend M.G. (2017). Chemosensitivity of *IDH1*-mutated gliomas due to an impairment in PARP1-mediated DNA repair. Cancer Res..

[75] Chan S.M., Thomas D., Corces-Zimmerman M.R., Xavy S., Rastogi S., Hong W.-J., Zhao F., Medeiros B.C., Tyvoll D.A., Majeti R. (2015). Isocitrate dehydrogenase 1 and 2 mutations induce BCL-2 dependence in acute myeloid leukemia. Nat. Med..

[76] Gelman S.J., Naser F., Mahieu N.G., McKenzie L.D., Dunn G.P., Chheda M.G., Patti G.J. (2018). Consumption of NADPH for 2-HG Synthesis Increases Pentose Phosphate Pathway Flux and Sensitizes Cells to Oxidative Stress. Cell Rep..

[77] Holmgren A., Lu J. (2010). Thioredoxin and thioredoxin reductase: Current research with special reference to human disease. Biochem. Biophys. Res. Commun..

[78] Fu X., Chin R.M., Vergnes L., Hwang H., Deng G., Xing Y., Pai M.Y., Li S., Ta L., Fazlollahi F. (2015). 2-hydroxyglutarate inhibits ATP synthase and mTOR Signaling. Cell Metab..

[79] Karpel-Massler G., Ishida C.T., Bianchetti E., Zhang Y., Shu C., Tsujiuchi T., Banu M.A., Garcia F., Roth K.A., Bruce J.N. (2017). Induction of synthetic lethality in *IDH1*-mutated gliomas through inhibition of Bcl-xL. Nat. Commun..

[80] Carbonneau M., Gagne L.M., Lalonde M.E., Germain M.A., Motorina A., Guiot M.C., Secco B., Vincent E.E., Tumber A., Hulea L. (2016). The oncometabolite 2-hydroxyglutarate activates the mTOR signalling pathway. Nat. Commun..

[81] Batsios G., Viswanath P., Subramani E., Najac C., Gillespie A.M., Santos R.D., Molloy A.R., Pieper R.O., Ronen S.M. (2019). PI3K/mTOR inhibition of *IDH1* mutant glioma leads to reduced 2HG production that is associated with increased survival. Sci. Rep..

[82] Wakimoto H., Tanaka S., Curry W.T., Loebel F., Zhao D., Tateishi K., Chen J., Klofas L.K., Lelic N., Kim J.C. (2014). Targetable Signaling Pathway Mutations Are Associated with Malignant Phenotype in *IDH*-Mutant Gliomas. Clin. Cancer Res..

[83] Fan Q.-W., Knight Z.A., Goldenberg D.D., Yu W., Mostov K.E., Stokoe D., Shokat K.M., Weiss W.A. (2006). A dual PI3 ki-nase/mTOR inhibitor reveals emergent efficacy in glioma. Cancer Cell.

[84] Puli S., Jain A., Lai J.C.K., Bhushan A. (2010). Effect of combination treatment of rapamycin and isoflavones on mtor pathway in human glioblastoma (U87) cells. Neurochem. Res..

[85] Sasaki M., Knobbe C.B., Itsumi M., Elia A.J., Harris I.S., Chio I.I.C., Cairns R.A., Mccracken S., Wakeham A., Haight J. (2012). D-2-hydroxyglutarate produced by mutant *IDH1* perturbs collagen maturation and basement membrane function. Genes Dev..

[86] Viswanath P., Radoul M., Izquierdo-Garcia J.L., Ong W.Q., Luchman H.A., Cairncross J.G., Huang B., Pieper R.O., Phil-lips J.J., Ronen S.M. (2018). 2-Hydroxyglutarate-Mediated Autophagy of the Endoplasmic Reticulum Leads To an Unusual Down-regulation of Phospholipid Biosynthesis in Mutant *IDH1* Gliomas. Cancer Res..

[87] Zhang Y., Pusch S., Innes J., Sidlauskas K., Ellis M., Lau J., El-Hassan T., Aley N., Launchbury F., Richard-Loendt A. (2019). Mutant *IDH* sensitizes gliomas to endoplasmic reticulum stress and triggers apoptosis via miR-183-mediated inhibition of semaphorin 3E. Cancer Res..

[88] Ou X., Liu Y., Lei X., Li P., Mi D., Ren L., Guo L., Guo R., Chen T., Hu J. (2020). Characterization of spike glycoprotein of SARS-CoV-2 on virus entry and its immune cross-reactivity with SARS-CoV. Nat. Commun..

[89] Yalaza C., Ak H., Cagli M.S., Ozgiray E., Atay S., Aydin H.H. (2017). R132H mutation in *IDH1* gene is associated with increased tumor HIF1-alpha and serum VEGF levels in primary glioblastoma multiforme. Ann. Clin. Lab. Sci..

[90] Jensen R.L. (2006). Hypoxia in the tumorigenesis of gliomas and as a potential target for therapeutic measures. Neurosurg. Focus.

[91] Gillespie D.L., Whang K., Ragel B.T., Flynn J.R., Kelly D.A., Jensen R.L. (2007). Silencing of hypoxia inducible factor-1α by RNA interference attenuates human glioma cell growth in vivo. Clin. Cancer Res..

[92] Fujiwara S., Nakagawa K., Harada H., Nagato S., Furukawa K., Teraoka M., Seno T., Oka K., Iwata S., Ohnishi T. (2007). Si-lencing hypoxia-inducible factor-1α inhibits cell migration and invasion under hypoxic environment in malignant gliomas. Int. J. Oncol..

[93] Tang J.H., Ma Z.X., Huang G.H., Xu Q.F., Xiang Y., Li N., Sidlauskas K., Zhang E.E., Lv S.Q. (2016). Downregulation of HIF-1a sensitizes U251 glioma cells to the temozolomide (TMZ) treatment. Exp. Cell Res..

[94] Kessler J., Hahnel A., Wichmann H., Rot S., Kappler M., Bache M., Vordermark D. (2010). HIF-1α inhibition by siRNA or chetomin in human malignant glioma cells: Effects on hypoxic radioresistance and monitoring via CA9 expression. BMC Cancer.

[95] Koivunen P., Lee S., Duncan C.G., Lopez G., Lu G., Ramkissoon S., Losman J.A., Joensuu P., Bergmann U., Gross S. (2012). Transformation by the (R)-enantiomer of 2-hydroxyglutarate linked to EGLN activation. Nature.

[96] Hartmann C., Hentschel B., Wick W., Capper D., Felsberg J., Simon M., Westphal M., Schackert G., Meyermann R., Pi-etsch T. (2010). Patients with *IDH1* wild type anaplastic astrocytomas exhibit worse prognosis than *IDH1*-mutated glioblas-tomas, and *IDH1* mutation status accounts for the unfavorable prognostic effect of higher age: Implications for classification of gliomas. Acta Neuropathol..

[97] Intlekofer A.M., DeMatteo R.G., Venneti S., Finley L.W.S., Lu C., Judkins A.R., Rustenburg A.S., Grinaway P.B., Chodera J.D., Cross J.R. (2015). Hypoxia Induces Production of L-2-Hydroxyglutarate. Cell Metab..

[98] Houillier C., Wang X., Kaloshi G., Mokhtari K., Guillevin R., Laffaire J., Paris S., Boisselier B., Idbaih A., Lai-gle-Donadey F. (2010). *IDH1* or *IDH2* mutations predict longer survival and response to temozolomide in low-grade glio-mas. Neurology.

[99] SongTao Q., Lei Y., Si G., YanQing D., HuiXia H., XueLin Z., LanXiao W., Fei Y. (2012). *IDH* mutations predict longer survival and response to temozolomide in secondary glioblastoma. Cancer Sci..

[100] van den Bent M.J., Dubbink H.J., Marie Y., Brandes A.A., Taphoorn M.J.B., Wesseling P., Frenay M., Tijssen C.C., Lacombe D., Idbaih A. (2010). *IDH1* and *IDH2* mutations are prognostic but not predictive for outcome in anaplastic oli-godendroglial tumors: A report of the European Organization for Research and Treatment of Cancer Brain Tumor Group. Clin. Cancer Res..

[101] Li S., Chou A.P., Chen W., Chen R., Deng Y., Phillips H.S., Selfridge J., Zurayk M., Lou J.J., Everson R.G. (2013). Overex-pression of isocitrate dehydrogenase 1/2 (*IDH1/2*) mutant protein renders glioma cells more sensitive to radiation. Neuro-Oncology.

[102] Tran A.N., Lai A., Li S., Pope W.B., Teixeira S., Harris R.J., Woodworth D.C., Nghiemphu P.L., Cloughesy T.F., El-lingson B.M. (2014). Increased sensitivity to radiochemotherapy in *IDH1* mutant glioblastoma as demonstrated by serial quantita-tive MR volumetry. Neuro-Oncology.

[103] Buckner J.C., Shaw E.G., Pugh S.L., Chakravarti A., Gilbert M.R., Barger G.R., Coons S., Ricci P., Bullard D., Brown P.D. (2016). Radiation plus Procarbazine, CCNU, and Vincristine in Low-Grade Glioma. N. Engl. J. Med..

[104] Sanson M., Marie Y., Paris S., Idbaih A., Laffaire J., Ducray F., el Hallani S., Boisselier B., Mokhtari K., Hoang-Xuan K. (2009). Isocitrate dehydrogenase 1 codon 132 mutation is an important prognostic biomarker in gliomas. J. Clin. Oncol..

[105] Claus E.B., Walsh K.M., Wiencke J.K., Molinaro A.M., Wiemels J.L., Schildkraut J.M., Bondy M.L., Berger M., Jenkins R., Wrensch M. (2015). Survival and low-grade glioma: The emergence of genetic information. Neurosurg. Focus..

[106] Wang Y., Wild A.T., Turcan S., Wu W.H., Sigel C., Klimstra D.S., Ma X., Gong Y., Holland E.C., Huse J.T. (2020). Target-ing therapeutic vulnerabilities with PARP inhibition and radiation in *IDH*-mutant gliomas and cholangiocarcinomas. Sci. Adv..

[107] Platten M., Bunse L., Wick A., Bunse T., le Cornet L., Harting I., Sahm F., Sanghvi K., Tan C.L., Poschke I. (2021). A vac-cine targeting mutant *IDH1* in newly diagnosed glioma. Nature.

[108] Hodges T.R., Ott M., Xiu J., Gatalica Z., Swensen J., Zhou S., Huse J.T., de Groot J., Li S., Overwijk W.W. (2017). Muta-tional burden, immune checkpoint expression, and mismatch repair in glioma: Implications for immune checkpoint im-munotherapy. Neuro-Oncology.

[109] Qazi M.A., Vora P., Venugopal C., Sidhu S.S., Moffat J., Swanton C., Singh S.K. (2017). Intratumoral heterogeneity: Pathways to treatment resistance and relapse in human glioblastoma. Ann. Oncol..

[110] Schumacher T., Bunse L., Pusch S., Sahm F., Wiestler B., Quandt J., Menn O., Osswald M., Oezen I., Ott M. (2014). A vac-cine targeting mutant *IDH1* induces antitumour immunity. Nature.

[111] Kohanbash G., Carrera D.A., Shrivastav S., Ahn B.J., Jahan N., Mazor T., Chheda Z.S., Downey K.M., Watchmaker P.B., Beppler C. (2017). Isocitrate dehydrogenase mutations suppress STAT1 and CD8+ T cell accumulation in gliomas. J. Clin. Invest..

[112] Berghoff A.S., Kiesel B., Widhalm G., Wilhelm D., Rajky O., Kurscheid S., Kresl P., Wöhrer A., Marosi C., Hegi M.E. (2017). Correlation of immune phenotype with *IDH* mutation in diffuse glioma. Neuro-Oncology.

[113] Amankulor N.M., Kim Y., Arora S., Kargl J., Szulzewsky F., Hanke M., Margineantu D.H., Rao A., Bolouri H., Delrow J. (2017). Mutant *IDH1* regulates the tumor-associated immune system in gliomas. Genes Dev..

[114] Bunse L., Pusch S., Bunse T., Sahm F., Sanghvi K., Friedrich M., Alansary D., Sonner J.K., Green E., Deumelandt K. (2018). Suppression of antitumor T cell immunity by the oncometabolite (R)-2-hydroxyglutarate. Nat. Med..

[115] Kadiyala P., Carney S.V., Gauss J.C., Garcia-Fabiani M.B., Haase S., Alghamri M.S., Núñez F.J., Liu Y., Yu M., Taher A.W. (2020). Inhibition of 2-Hydroxyglutarate Elicits Metabolic-reprograming and Mutant *IDH1* Glioma Immunity in Mice. J. Clin. Invest..

[116] Pellegatta S., Valletta L., Corbetta C., Patanè M., Zucca I., Sirtori F.R., Bruzzone M.G., Fogliatto G., Isacchi A., Pollo B. (2015). Effective immuno-targeting of the *IDH1* mutation R132H in a murine model of intracranial glioma. Acta Neuropathol. Commun..

[117] Platten M., Schilling D., Bunse L., Wick A., Bunse T., Riehl D., Green E., Sanghvi K., Karapanagiotou-Schenkel I., Hart-ing I. (2018). ATIM-33. NOA-16: A first-in-man multicenter phase i clinical trial of the german neurooncology working group evaluating a mutation-specific peptide vaccine targeting idh1r132h in patients with newly diagnosed malignant as-trocytomas. Neuro-Oncology.

[118] Sampson J.H., Choi B.D., Sanchez-Perez L., Suryadevara C.M., Snyder D.J., Flores C.T., Schmittling R.J., Nair S.K., Reap E.A., Norberg P.K. (2014). EGFRvIII mCAR-modified T-cell therapy cures mice with established intracerebral glioma and generates host immunity against tumor-antigen loss. Clin. Cancer Res..

[119] O’Rourke D.M., Nasrallah M.P., Desai A., Melenhorst J.J., Mansfield K., Morrissette J.J.D., Martinez-Lage M., Brem S., Maloney E., Shen A. (2017). A single dose of peripherally infused EGFRvIII-directed CAR T cells mediates antigen loss and induces adaptive resistance in patients with recurrent glioblastoma. Sci. Transl. Med..

[120] Senhaji N., Louati S., Chbani L., el Fatemi H., Hammas N., Mikou K., Maaroufi M., Benzagmout M., Boujraf S., el Bardai S. (2017). EGFR Amplification and *IDH* Mutations in Glioblastoma Patients of the Northeast of Morocco. Biomed Res. Int..

[121] Taher M.M., Dairi G., Butt E.M., Al-Quthami K., Al-Khalidi H., Jastania R.A., Nageeti T.H., Bogari N.M., Athar M., Al-Allaf F.A. (2020). EGFRvIII expression and isocitrate dehydrogenase mutations in patients with glioma. Oncol. Lett..

[122] Flavahan W.A., Drier Y., Liau B.B., Gillespie S.M., Venteicher A.S., Stemmer-Rachamimov A.O., Suvà M.L., Bernstein B.E. (2016). Insulator dysfunction and oncogene activation in *IDH* mutant gliomas. Nature.

[123] Xiao W., Wang J., Wen X., Xu B., Que Y., Yu K., Xu L., Zhao J., Pan Q., Zhou P. (2020). Chimeric antigen recep-tor-modified T-cell therapy for platelet-derived growth factor receptor α-positive rhabdomyosarcoma. Cancer.

[124] Borodovsky A., Salmasi V., Turcan S., Fabius A.W.M., Baia G., Eberhart C.G., Weingart J.D., Gallia G.L., Baylin S.B., Chan T.A. (2013). 5-azacytidine reduces methylation, promotes differentiation and induces tumor regression in a pa-tient-derived *IDH1* mutant glioma xenograft. Oncotarget.

[125] Yamashita A.S., Rosa M.d., Borodovsky A., Festuccia W.T., Chan T., Riggins G.J. (2019). Demethylation and epigenetic modifica-tion with 5-azacytidine reduces *IDH1* mutant glioma growth in combination with temozolomide. Neuro-Oncology.

[126] Oganesian A., Redkar S., Taverna P., Joshi-Hangal R., Azab M. (2013). Preclinical data in cynomolgus (cyn) monkeys of ASTX727, a novel oral hypomethylating agent (HMA) composed of low-dose oral decitabine combined with a novel cytidine deami-nase inhibitor (CDAi) E7727. Blood.

[127] Garcia-Manero G., Griffiths E.A., Steensma D.P., Roboz G.J., Wells R., McCloskey J., Odenike O., DeZern A.E., Yee K., Busque L. (2020). Oral cedazuridine/decitabine for MDS and CMML: A phase 2 pharmacokinetic/pharmacodynamic random-ized crossover study. Blood.

[128] Bai H., Harmancı A.S., Erson-Omay E.Z., Li J., Coşkun S., Simon M., Krischek B., Özduman K., Omay S.B., Sorensen E.A. (2016). Integrated genomic characterization of *IDH1*-mutant glioma malignant progression. Nat. Genet..

